# Neural effects of acupuncture on stroke patients with motor dysfunction: an activation likelihood estimation meta-analysis

**DOI:** 10.3389/fneur.2024.1453935

**Published:** 2024-09-25

**Authors:** Dongxia Li, Dongyan Wang, Yihao Zhou, Yuan Zhang, Siyu Yang, Xu Dong, Shaojie Cai, Ruiting Zhang

**Affiliations:** ^1^The Second Clinical Medical College of Heilongjiang University of Traditional Chinese Medicine, Harbin, China; ^2^The Second Affiliated Hospital of Heilongjiang University of Chinese Medicine, Harbin, China

**Keywords:** motor dysfunction after stroke, activation likelihood estimation, functional magnetic resonance imaging, meta-analysis, acupuncture

## Abstract

**Background:**

Functional magnetic resonance imaging has been used in many studies to explore the neural mechanism of acupuncture in patients with post-stroke motor dysfunction. Inconsistent results have been found in these studies, however. This activation likelihood estimation (ALE) meta-analysis was designed to quantitatively integrate changes in brain activity and the neurological effects of acupuncture on patients with motor dysfunction after stroke.

**Methods:**

We searched PubMed, Embase, Web of Science, the Cochrane Library, China Science and Technology Journal Database, the China Biology Medicine, the China National Knowledge Infrastructure, and Wanfang Data Knowledge Service Platform for literature from the establishment of the database until March 21, 2024. Research papers were selected, data extracted, and quality assessment was done independently by two researchers. The GingerALE software was used for meta-analysis, and Jackknife sensitivity analysis was employed to assess result robustness.

**Results:**

We ended up analyzing 14 studies that included 235 patients and 100 healthy people. ALE meta-analysis showed that Compared with healthy people, the enhanced brain region in poststroke patients with motor dysfunction was located in the left posterior lobe of the cerebellum, the left inferior frontal gyrus, and the left precuneus (*p* < 0.001). After acupuncture, the activated regions were mainly located in the left posterior lobe of the cerebellum, the right lentiform nucleus putamen, the right medial frontal gyrus, the right inferior frontal gyrus, the left precuneus, the right middle temporal gyrus, the left claustrum, the left cerebellar tonsil, the right superior marginal gyrus, the inactivated area is located in the right medial frontal gyrus the left precentral gyrus and the right precuneus (*p* < 0.001).

**Conclusion:**

Acupuncture can reestablish motor function by causing extensive changes in the brain function of patients with stroke, which may be the neurological effect of acupuncture therapy on stroke patients.

**Systematic review registration:**

https://www.crd.york.ac.uk/prospero/, identifier CRD42024526263.

## 1 Introduction

Stroke is an acute brain injury caused by rupture or accidental blockage of a cerebral blood vessel, which is characterized by high morbidity, disability, mortality, and recurrence (1), and is the world’s leading cause of death and the third leading cause of disability (2). After stroke, about 70 to 80% of patients cannot live independently due to disability (3), which often increases the financial burden of patients and their families, resulting in post-stroke depression, anxiety, and other emotions. The reconstruction of limb movement could ease the financial burden of stroke care.

Chinese medicine therapy, including acupuncture, massage, moxibustion, and other traditional techniques, is widely recognized and utilized in the rehabilitation of stroke hemiplegia. Acupuncture, one of the distinctive techniques of traditional Chinese medicine, has been widely used in the rehabilitation of hemiplegia after stroke and is recommended by the World Health Organization as an alternative and complementary strategy for stroke treatment (4). More and more studies have confirmed that acupuncture has a clear therapeutic effect in promoting the reconstruction of nerve function and the repair of limb motor function (5). In addition, American Stroke Association guidelines also indicate that acupuncture promotion can be effective as an adjunct to motor recovery and walking activities (6).

A resting-state functional magnetic resonance imaging (fMRI) technique measures spontaneous neuronal activity by capturing signals related to blood oxygen levels (BOLD). Amplitude of low frequency fluctuations (ALFF), which reflects the intensity of regional fluctuations in the BOLD signal, and regional homogeneity (ReHo), which measures BOLD signal coherence across neighboring voxels and a single voxel. Hence, ALFF and ReHo together are adequate to show spontaneous brain activity (7–9). In order to better understand the neurological effects of acupuncture therapy, an increasing number of acupuncture studies are applying rs-fMRI to clinical trials. It has been found that the mechanism by which acupuncture can restore motor function in patients with post-stroke functional dysfunction is closely related to the regulation of brain function (5, 10). However, due to the small sample size or the differences in experimental design between studies, the results of various studies are different, which makes the neural mechanism of acupuncture therapy to improve motor function in patients controversial and the results difficult to generalize (11, 12). Therefore, there is a need to quantitatively integrate these discrepant results through meta-analysis.

Activation likelihood estimation (ALE) is a coordinates-based meta-analysis method. In recent years, ALE has been widely used in the meta-analysis of brain functions in the field of cognition. The ALE method performs three-dimensional Gaussian processing and statistical tests on the activation points of the included literature (13). To locate brain regions with statistical significance. Although previous literature applied Seedbased d Mapping with Permutation of Subject Images to quantitatively meta-analyze the changes of brain function in patients with post-stroke motor dysfunction with two treatments (14), However, the differences in brain regions between patients and healthy people were not studied, and the number of literatures included in the study was small. The purpose of this study is to use ALE algorithm to explore the brain regions related to acupuncture in patients with post-stroke motor dysfunction, which helps clarify the neural mechanism of acupuncture in patients with post-stroke motor dysfunction and may provide new ideas for acupuncture treatment of other neurodegenerative diseases.

## 2 Materials and methods

This ALE-meta analysis protocol was registered on PROSPERO and can be accessed through (registration number: CRD42024526263). This study followed the PRISMA guidelines, which recommend preferred reporting items for systematic reviews and meta-analyses (15).

### 2.1 Retrieval strategies

Eight databases, namely Web of Science, PubMed, Embase, the Cochrane Library, China Science and Technology Journal Database, the China Biology Medicine, the China National Knowledge Infrastructure and Wanfang Data Knowledge Service Platform, were performed a methodical and comprehensive search from their creation to March 21, 2024 by two authors (LDX and ZY). In addition, another researcher (ZYH) arbitrated inconsistent search results. The following search terms in a “subject plus free word” search strategy were used: (“stroke” OR “Cerebrovascular” OR “Poststroke” OR “Cerebrovascular Accident” OR “CVA” OR “Cerebrovascular Apoplexy” OR “Brain Vascular Accident” OR “Cerebrovascular Stroke” OR “Apoplexy” OR “Cerebral Stroke” OR “Acute Stroke” OR “Acute Cerebrovascular Accident” OR “hemiplegia” OR “Cerebral Infarction”) AND (“acupuncture” OR “electroacupuncture” OR “needle” OR “acupoint” OR “pharmacopuncture”) AND (“fMRI” OR “functional MRI” OR “functional magnetic resonance imaging” OR “neuroimaging” OR “resting-state functional magnetic resonance” OR “rs-fMRI”). For detailed search strategies, see the Supplementary material. In addition, references in these articles were manually reviewed to ensure that no relevant literature was omitted.

### 2.2 Study selection

Endnote (X9) was used to import the retrieved studies. After eliminating duplicate studies using the software, two authors (LDX and ZY) reviewed the title and abstract according to the inclusion and exclusion criteria to exclude the obviously non-conforming articles, and then reviewed the full text for screening.

#### 2.2.1 Inclusion criteria

1. Any patient meeting the diagnostic criteria for motor dysfunction after stroke established by a clinical guideline or consensus of international repute such as that of the Chinese Medical Association;
2. Including both randomized and non-randomized controlled trials;
3. A variety of forms of acupuncture were used (manual acupuncture, electroacupuncture); manipulation methods, acupoint selection, and duration of acupuncture were not restricted;
4. In the studies, neuroimaging results (ALFFs or ReHo) were compared (between acupuncture patients and healthy controls) via fMRI, utilizing the standard anatomical template (Talairach or Montreal Neurological Institute [MNI]) in three dimensions (x, y, and z).

#### 2.2.2 Exclusion criteria

1. The results were based on partial coverage or employing only region of interest;
2. Reviews, animal experiments and secondary studies;
3. No available stereotactic peak coordinates through various approaches;
4. Full texts were unavailable through extensive search;
5. Multiple articles using the same data include only one of them.

### 2.3 Data extraction

Two reviewers (LDX and ZY) independently extracted and checked the following items. The information included: author name, year of publication, type of study, comparisons, sample size, subject characteristics, acupuncture methods and neuroimaging data (MRI acquisition, processing parameters, analysis parameters, activation coordinates, deactivation coordinate and standard anatomical template). In case of disagreement, a third reviewer (ZYH) will resolve differences between researchers.

### 2.4 Quality assessments

Two reviewers (LDX and ZY) evaluated the quality of the included studies according to methodological index for non-randomized studies 5 (MINORS5) (16) and the Cochrane risk of bias tools.^1^ Non-randomized controlled trials are evaluated using MINORS5, which has 12 items. We used risk-of-bias tools developed by the Cochrane risk of bias tools with seven different factors to assess the quality of randomized controlled trials. A third reviewer (ZYH) resolved the discrepancies.

### 2.5 ALE meta-analysis

GingerALE software (version 3.0.2) was used for the coordinate-based ALE meta-analysis. Firstly, the coordinates reported in the research were unified into file data and all were unified into MNI space coordinates using Ginger ALE software. We set the parameters as threshold settings of uncorrected *p* value <0.001 and a minimum volume of 400 mm^3^. Finally, the Mango software is used to view the ALE result threshold graph obtained from the statistical test.

### 2.6 Sensitivity analysis

We employed Jackknife sensitivity analysis to assess the replicability of ALE meta-analysis findings, wherein each study is systematically excluded and the remaining studies are reevaluated (17, 18).

## 3 Results

### 3.1 Search selection

**Figure 1:**
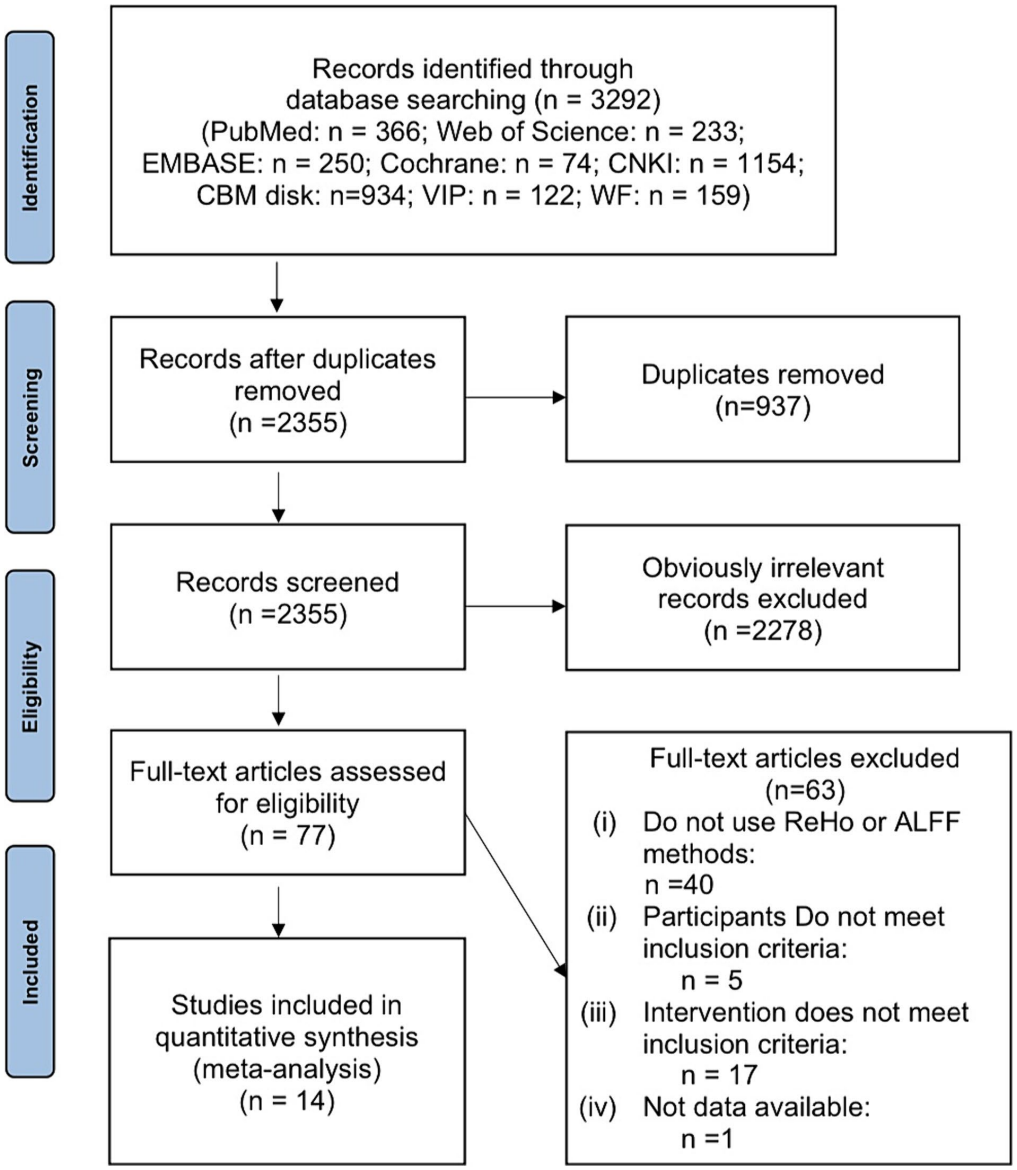
Flow diagram of literature search.

In Figure 1, a detailed explanation of the screening process is shown. It was possible to retrieve 3,292 articles in total. After the removal of 937 duplicate papers, there remained 2,355 articles. Upon review of titles and abstracts, 2,278 studies that were clearly irrelevant were excluded. Following a thorough examination of the full text, 63 records were excluded based on the developed inclusion and exclusion criteria. Ultimately, the remaining 14 studies were deemed suitable for inclusion in this meta-analysis.

### 3.2 Characteristics of included studies

We identified 14 eligible studies (11, 12, 19–30), 2 in English (11, 29) and 12 in Chinese (12, 19–28, 30). Table 1 provides details of all included studies. There were eight randomized controlled trials (11, 19, 21–24, 26, 28) and six non-randomized controlled trials (12, 20, 25, 27, 29, 30) involving 235 patients and 100 healthy people. Six of the studies (12, 19, 20, 22, 25, 27) recruited healthy controls, and the demographics of all the included healthy controls were matched to the demographics of the patients in each study. Acupuncture intervention was used in all study treatment groups, except for electroacupuncture in three studies (12, 21, 28), the other intervention methods were hand acupuncture. In terms of analytical methods, fMRI was used to measure ReHo and ALFF in all studies, including 11 studies (11, 19–24, 26–29) using ReHo and 3 studies (12, 25, 30) using ALFF. Sixteen studies provided data on changes in the brain regions of patients before and after acupuncture, and six trials provided comparisons of brain regions between patients and healthy controls. Neuroimaging data were obtained at 1.5 T(3 studies) (23, 25, 26) or 3 T (9 studies) (11, 12, 19, 22, 24, 27–30), while two studies (20, 21) did not specify magnetic field strength. Six studies (11, 12, 24, 27, 28, 30) used Siemens MRI scanners, six studies (19, 22, 23, 25, 26, 29) used General Electric, and two studies (20, 21) did not report scanners. All studies were whole brain scans. An analysis of fMRI data is provided in Table 2.

**Table 1:** Characteristics of included studies.

Study	Disease	Study type	Sample size		Age (years ± SD)		Gender (male/female)		Comparison		Regimen	Experiments	Outcome measures	
			AG	CG Patient/HC	Patient	Patient/HC	Patient	Patient/HC	T	C			Analysis of fMRI	Secondary outcomes
Jianzhong (30)	Poststroke limb motion dysfunction	Non-RCT	8	–	62 ± 9.44	–	8/0	–	MA	None	2 weeks	1	ALFF	FMA
Zhihong et al. (29)	Poststroke limb motion dysfunction	Non-RCT	10	–	57.70 ± 7.69	–	7/3	–	MA	None	15 min	1	ReHo	Deqi
Ximei et al. (28)	Poststroke limb motion dysfunction	RCT	6	6	63.20 ± 1.34	65.25 ± 1.89	3/3	4/2	EA	MA	6 weeks	2	ReHo	–
Fang-yuan et al. (12)	Poststroke limb motion dysfunction	Non-RCT	33	30	62.8 ± 1.65	63.4 ± 2.69	18/15	17/13	EA	HC	4 weeks	1	fALFF	BI
Caihong et al. (27)	Poststroke limb motion dysfunction	Non-RCT	20	20	61.32 ± 8.53	58.86 ± 8.53	13/7	10/10	MA	HC	10 min	2	ReHo	–
Zhengfei et al. (26)	Poststroke limb motion dysfunction	RCT	42	42	55.67 ± 8.38	57.01 ± 6.97	22/20	24/18	MA	ConV	2 weeks	1	ReHo	NDS, BI, FMA
Cai-xia et al. (25)	Poststroke limb motion dysfunction	Non-RCT	10	10	68.80 ± 2.70	67.80 ± 2.90	6/4	5/5	MA	HC	30 days	1	ALFF	–
Nana (24)	Poststroke limb motion dysfunction	RCT	6	6	62.00 ± 8.4851	67.33 ± 8.779	2/4	4/2	MA	SA	10 days	2	ReHo	FMA, MBI
Ping et al. (11)	ischemic stroke	RCT	11	10	69.364 ± 8.119	61.300 ± 7.938	7/4	5/5	MA	ConV	4 weeks	1	ReHo	NDS, MBI, FMA
Zhihong (23)	Poststroke limb motion dysfunction	RCT	30	30	62.67 ± 3.79	65.33 ± 5.31	17/13	18/12	MA(SYTD)	MA(XNKQ)	14 days	2	ReHo	BI, FMA
Yumei (22)	Poststroke limb motion dysfunction	RCT	11	15	69.36 ± 4.06	62.07 ± 5.6	7/4	9/6	MA	HC	4 weeks	2	ReHo	NDS, FMA, ADL
Hinjing (21)	Poststroke limb motion dysfunction	RCT	12	12	59.05 ± 7.39	60.87 ± 6.47	5/7	6/6	EA	MA	6 weeks	2	ReHo	NDS, BI, FMA
Xie et al. (20)	Poststroke limb motion dysfunction	Non-RCT	9	10	65.50 ± 2.32	66.20 ± 2.70	5/4	6/4	MA	HC	4 weeks	2	ReHo	–
Xiaoqing (19)	Poststroke limb motion dysfunction	RCT	11	15	64.45 ± 3.86	62.07 ± 2.61	7/4	9/6	MA	HC	4 weeks	2	ReHo	NDS, FMA, MBI

RCT, randomized controlled trial; Non-RCT, non-randomized controlled trial; ALFF, amplitude of low-frequency fluctuation; fALFF, fractional amplitude of low-frequency fluctuation; ReHo, regional homogeneity; AG, acupuncture group; CG, control group; T, treatment group; C, control group; fMRI, functional magnetic resonance imaging; MA, manual acupuncture; EA, electro-acupuncture; Conv, conventional treatment; HC, health control; FMA, fugl-meyer assessment; BMI, modi ed barthel index; BI, barthel index; NDS, neuropathy disability score; SYTD, shengyang tongdu needling method; XNKQ, xingnao kaiqiao needling method.

**Table 2:** The details of MRI acquisition and analysis.

Study	MRI acquisition		Seed regions	Threshold	Number of coordinates	Analysis	
	Teslas	MRI-system				Software	Method
Jianzhong (30)	3.0 T	Siemens	Whole brain	*p* < 0.05, uncorrected	MNI,5	SPM12	ALFF
Zhihong et al. (29)	3.0 T	GE	Whole brain	–	MNI,5	SPM8	ReHo
Ximei et al. (28)	3.0 T	Siemens	Whole brain	–	Talairach,19/11	–	ReHo
Fang-yuan et al. (12)	3.0 T	Siemens	Whole brain	*p* < 0.01, AlphaSim corrected	MNI,11	SPM12	fALFF
Caihong et al. (27)	3.0 T	Siemens	Whole brain	*p* < 0.02, monte carlo	Talairach,15/6	SPM8	ReHo
Zhengfei et al. (26)	1.5 T	GE	Whole brain	*p* < 0.05, uncorrected	MNI,5	SPM	ReHo
Cai-xia et al. (25)	1.5 T	GE	Whole brain	*p* < 0.05, uncorrected	Talairach,11	SPM	ALFF
Nana (24)	3.0 T	Siemens	Whole brain	*p* < 0.05, uncorrected	MNI,9/5	SPM8	ReHo
Ping et al. (11)	3.0 T	Siemens	Whole brain	*p* < 0.05, uncorrected	Talairach,8	SPM2	ReHo
Zhihong (23)	1.5 T	GE	Whole brain	*p* < 0.05, uncorrected	MNI,5/5	SPM	ReHo
Yumei (22)	3.0 T	GE	Whole brain	*p* < 0.05, uncorrected	MNI,7/22	SPM5	ReHo
Hinjing (21)	–	–	Whole brain	*p* < 0.05, uncorrected	Talairach,19/11	SPM	ReHo
Xie et al. (20)	–	–	Whole brain	*p* < 0.05, uncorrected	Talairach,20/12	–	ReHo
Xiaoqing (19)	3.0 T	GE	Whole brain	*p* < 0.01, uncorrected	MNI,11/19	SPM5	ReHo

MRI, magnetic resonance imaging; GE, gradient echo pulse; SPM, statistical parametric mapping; fALFF, fractional amplitude of low-frequency fluctuation; ALFF, amplitude of low-frequency fluctuation; ReHo, regional homogeneity; MNI, montreal neurological institute.

### 3.3 Quality assessment

**Figure 2:**
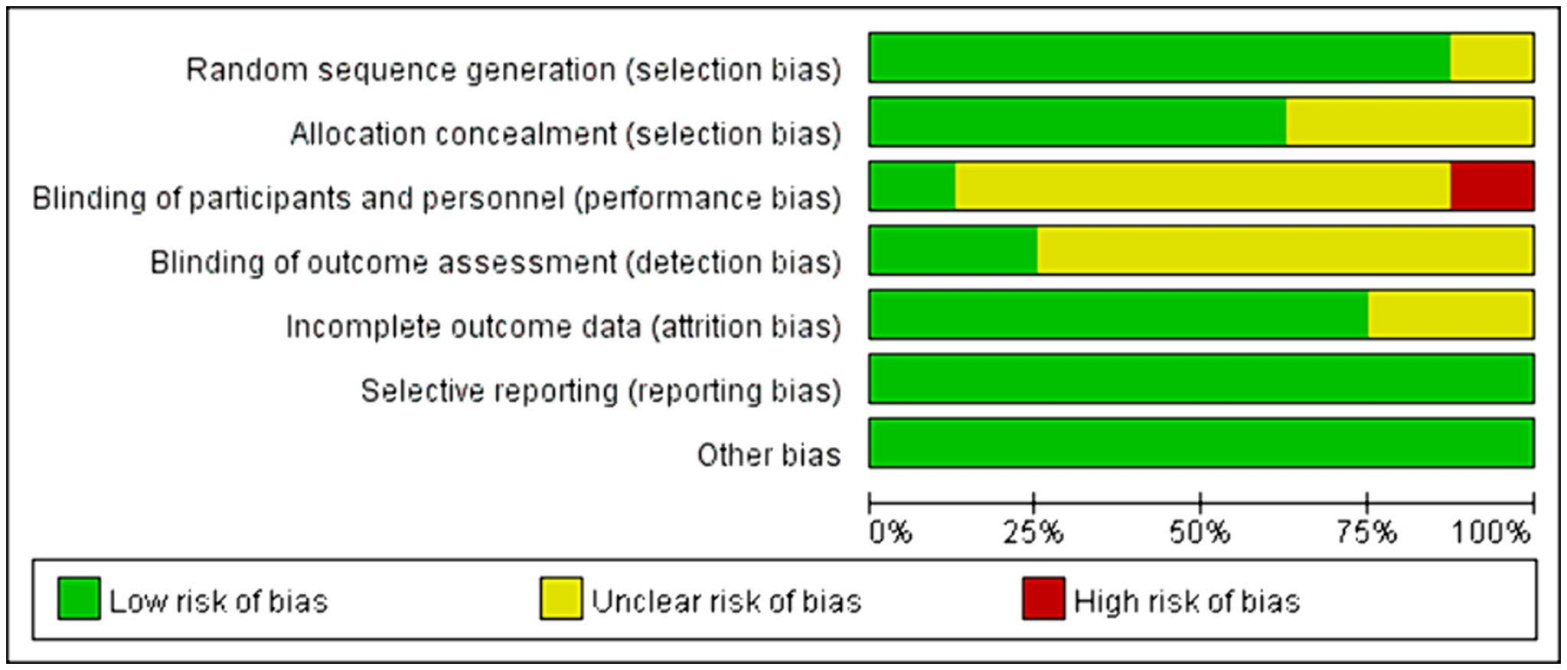
Risk of bias summary.

Among all the eligible studies, 8 were randomized controlled trials (11, 19, 21–24, 26, 28), and the Cochrane risk bias tool was utilized for quality assessment, consisting of a total of 7 evaluation criteria. Seven of the articles provided detailed descriptions of the randomization process, with two (11, 21) utilizing computer software randomization and five (19, 22–24, 26) using the random number table method. One study (28) mentioned “random” without specifying further details. Five studies (11, 19, 21, 23, 24) employed sealed envelopes for allocation concealment, while three (22, 26, 28) did not mention any allocation hiding methods. Only one study (19) used a triple-blind approach and the rest did not discuss blinding or allocation hiding, resulting in high performance bias levels. Some missing data led to high attrition bias. There was no evidence of selective reporting bias in any of the literature and it was rated as “low risk.” No other significant risks were identified in any of the literature, so other biases were evaluated as “low risk.” Specific information on bias assessment is presented in Figure 2. The remaining 6 non-randomized controlled trials (12, 20, 25, 27, 29, 30) were evaluated by MINORS5, and the results were shown in Table 3.

**Table 3:** Risk of bias summary by MINORS5.

Number	Entries	Study	Jianzhong (30)	Chen et al. (29)	Fang-yuan et al. (12)	Caihong et al. (27)	Cai-xia et al. (25)	Xie et al. (20)
1	A clearly stated aim		2	2	2	2	2	2
2	Inclusion of consecutive patients		2	2	2	2	2	2
3	Prospective collection of data		2	2	2	2	2	2
4	Endpoints appropriate to the aim of the study		2	2	2	2	2	2
5	Unbiased assessment of the study endpoint		1	1	1	2	2	2
6	Follow-up period appropriate to the aim of the study		0	0	0	0	0	0
7	Loss to follow up less than 5%		0	0	0	0	0	0
8	Prospective calculation of the study size		0	0	0	0	0	0
Additional criteria in the case of comparative studys								
9	An adequate control group				2	2	2	2
10	Contemporary groups				2	2	2	2
11	Baseline equivalence of groups				0	2	2	2
12	Adequate statistical analyses				2	2	2	2
	**Aggregate score**		**9**	**9**	**15**	**18**	**18**	**18**

The items are scored 0 (not reported), 1 (reported but inadequate) or 2 (reported and adequate). The global ideal score being 16 for non-comparative studies and 24 for comparative studies.

### 3.4 ALE meta-analysis results

#### 3.4.1 A comparison of brain neural activity in patients with motor dysfunction after stroke and HC

**Figure 3:**
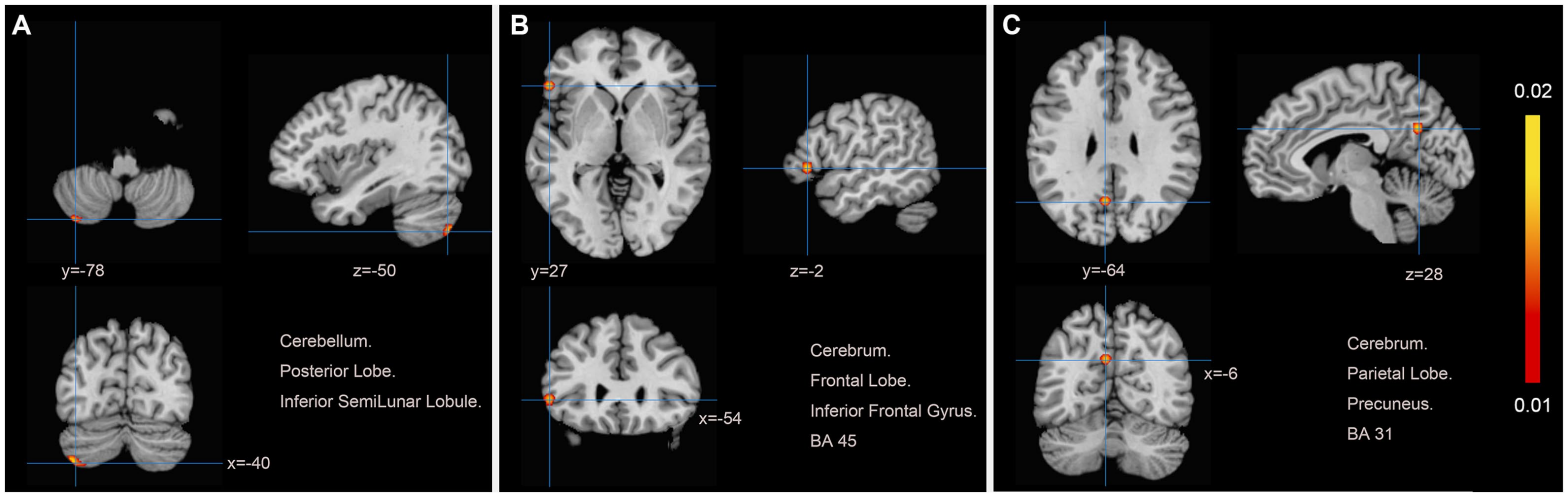
Patients with motor dysfunction after stroke have increased brain spontaneous activity relative to HCs. **(A)** Cerebellum Posterior Lobe Inferior SemiLunar Lobule; **(B)** Cerebrum Frontal Lobe Inferior Frontal Gyrus BA45; **(C)** Cerebrum Parietal Lobe Precuneus BA31.

Data from six studies (12, 19, 20, 22, 25, 27) assessed changes in brain region function in patients with motor dysfunction after stroke compared to healthy people. We extracted 61 brain activation regions from six trials and 14 brain inactivation regions from four experiments. Compared with healthy people, we found increased activity in three brain regions in patients with poststroke motor dysfunction:(1) the left posterior lobe of the cerebellum; (2) the left inferior frontal gyrus (IFG); (3) the left precuneus (PCUN), and no brain regions with reduced activity were found. Table 4 and Figure 3 provide specific analysis results.

**Table 4:** Changes of brain activation.

Number	Volume (mm^3^)	MNI coordinates			Peak ALE value	Hemisphere	Brain regions		Brodmann area	*p*
Patients with motor function after stroke > Healthy person										
1	640	−40	−78	−50	0.018689122	L	Cerebellum Posterior Lobe Inferior SemiLunar Lobule	–	–	0.00000004071122
2	464	−54	27	−2	0.016297676	L	Frontal Lobe Inferior Frontal Gyrus	IFG	45	0.00000056572526
3	432	−6	−64	28	0.01622	L	Parietal Lobe Precuneus	PCUN	31	0.00000063132217
Post-acupuncture > Pre-acupuncture										
1	1,184	−34	−78	−38	0.015235205	L	Cerebellum Posterior Lobe Inferior SemiLunar Lobule	–	–	0.0000005667985
2	960	34	−8	8	0.016314756	R	Sub-lobar Lentiform Nucleus Putamen	PUT	–	0.00000019121583
3	904	28	50	4	0.017546684	R	Frontal Lobe Medial Frontal Gyrus	MFG	9	0.000000053811792
4	856	50	18	24	0.017312586	R	Frontal Lobe Inferior Frontal Gyrus	IFG	9	0.00000006894088
5	616	−28	−64	34	0.012985027	L	Parietal Lobe Precuneus	PCUN	7	0.0000054770453
6	472	70	−22	−16	0.014600864	R	Temporal Lobe Middle Temporal Gyrus	MTG	21	0.0000010888054
7	448	−34	−6	10	0.014138335	L	Sub-lobar Claustrum	CLA	-	0.0000017363611
8	408	−30	−64	−46	0.012140173	L	Cerebellum Posterior Lobe Cerebellar Tonsil	–	–	0.000012553858
9	400	54	−38	36	0.012090526	R	Parietal Lobe Supra marginal Gyrus	SMG	40	0.000013195734
Post-acupuncture < Pre-acupuncture										
1	960	18	10	62	0.014834693	R	Frontal Lobe Medial Frontal Gyrus	MFG	6	0.000000036710198
2	688	−24	−18	60	0.01341833	L	Frontal Lobe Precentral Gyrus	PreCG	4	0.00000023600485
3	472	4	−58	64	0.008947058	R	Parietal Lobe Precuneus	PCUN	7	0.000038893137

IFG, inferior frontal gyrus; PCUN, precuneus; PUT, lentiform nucleus putamen; MFG, medial frontal gyrus; MTG, middle temporal gyrus; CLA, claustrum; SMG, superior marginal gyrus; PreCG, precentral gyrus; R, right; L, left.

#### 3.4.2 Acupuncture manipulation of brain areas in patients with motor dysfunction after stroke

**Figure 4:**
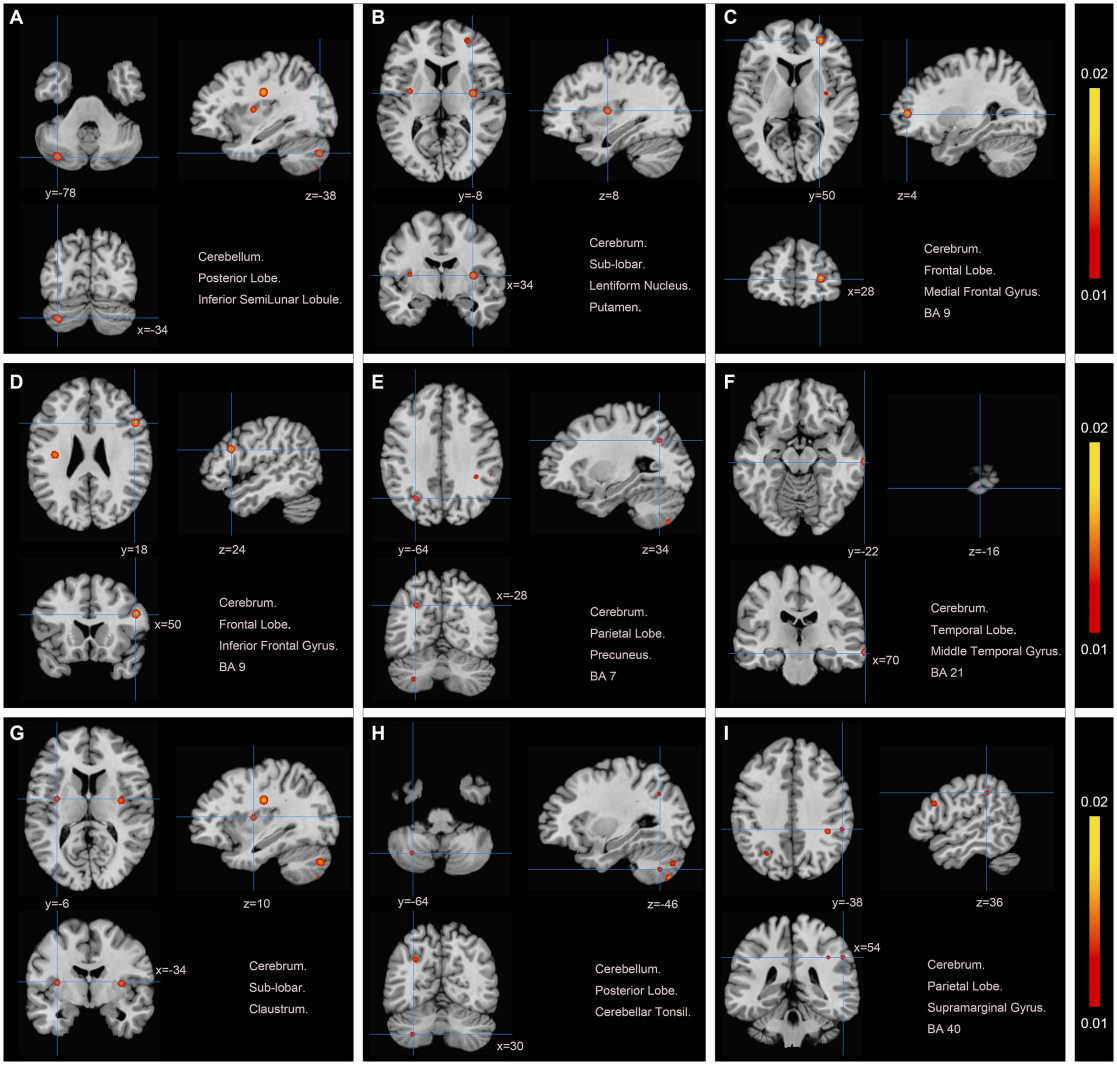
Regions of brain activation in patients with pre-to post-acupuncture (Post-acupuncture > Pre-acupuncture) **(A)** Cerebellum Posterior Lobe Inferior SemiLunar Lobule; **(B)** Cerebrum Sub-lobar Lentiform Nucleus Putamen; **(C)** Cerebrum Frontal Lobe Medial Frontal Gyrus BA9; **(D)** Cerebrum Frontal Lobe Inferior Frontal Gyrus BA9; **(E)** Cerebrum Parietal Lobe Precuneus BA7; **(F)** Cerebrum Temporal Lobe Middle Temporal Gyrus BA21; **(G)** Cerebrum Sub-lobar Claustrum; **(H)** Cerebellum Posterior Lobe Cerebellar Tonsil; **(I)** Cerebrum Parietal Lobe Supra marginal Gyrus BA40.

**Figure 5:**
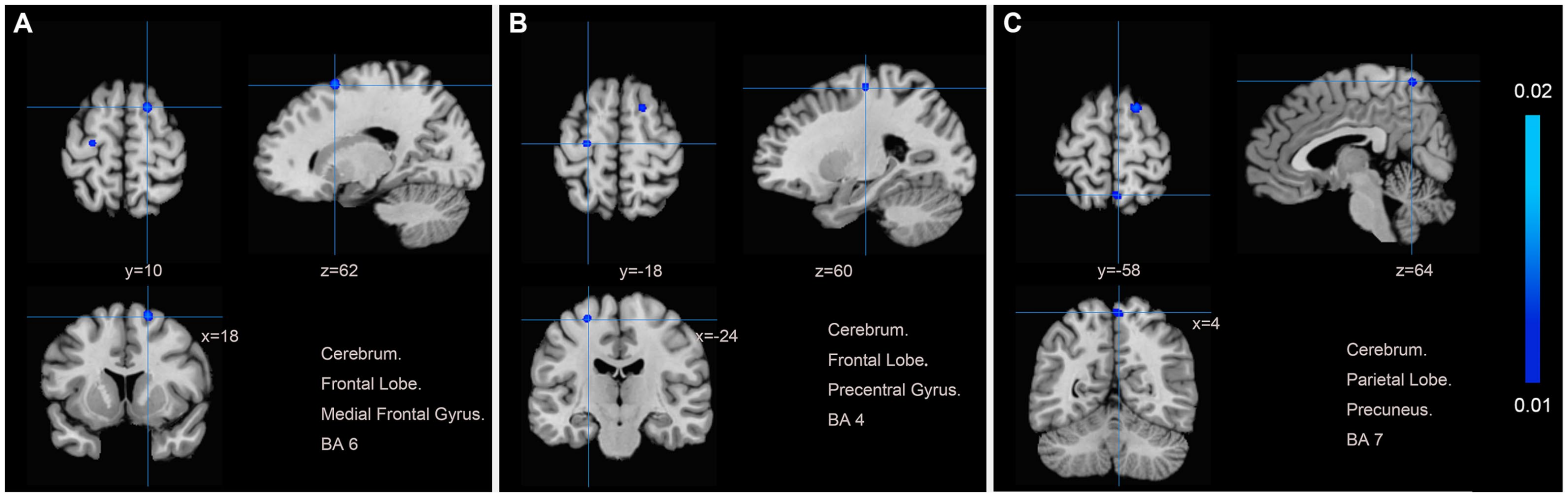
Regions of brain activation in patients with pre-to post-acupuncture (Post-acupuncture < Pre-acupuncture) **(A)** Cerebrum Frontal Lobe Medial Frontal Gyrus BA6; **(B)** Cerebrum Frontal Lobe Precentral Gyrus BA4; **(C)** Cerebrum Parietal Lobe Precuneus BA7.

We pooled data from 12 studies (11, 19–24, 26–30) to investigate the neurological effects of acupuncture on patients with post-stroke motor dysfunction. We extracted 133 brain activation areas from 16 trials and 45 brain inactivation areas from 10 trials. A total of 9 activation clusters were found before and after acupuncture:(1) the left posterior lobe of the cerebellum; (2) the right lentiform nucleus putamen (PUT); (3) the right medial frontal gyrus (MFG); (4) the right IFG; (5) the left PCUN; (6) the right middle temporal gyrus (MTG); (7) the left claustrum (CLA); (8) the left cerebellar tonsil; (9) the right superior marginal gyrus (SMG). Three inactivated clusters were detected before and after acupuncture: (1) the right MFG; (2) the left precentral gyrus (PreCG); (3) the right PCUN. Figures 4, 5 and Table 4 show the specific analysis results.

### 3.5 Sensitivity analysis

We re-sampled the included studies using the Jackknife method. Compared with healthy people, there were a total of 6 original studies on the enhancement of cerebral nerve activity in patients. The analysis results showed that the repeatability of the left posterior cerebellar lobe reached 3 times, and the repeatability of IFG_L and PCUN_L reached 4 times. There were 16 original tests on brain region activation of patients after acupuncture. Analysis showed that the repeatability of the left posterior cerebellar lobe reached 16 times, the repeatability of PUT_R reached 15 times, and the repeatability of MFG_R, IFG_R, CLA_L and Cerebellar Tonsil_L all reached 14 times. PCUN_L repeatability reached 13 times. The repeatability of MTG_R and SMG_R both reached 12 times. There were 10 original trials of reduced brain activity in patients after acupuncture, and the results showed that PCUN_R repeatability was up to 9 times, and MFG_R and PreCG_L repeatability was up to 8 times (Table 5).

**Table 5:** Sensitive analyses.

Discarded Article	Patients with motor function after stroke > Healthy person		
	Posterior lobe of the cerebellum_L	IFG_L	PCUN_L
Xiaoqing (19)	N	N	N
Xie et al. (20)	N	Y	Y
Yumei (22)	Y	N	N
Cai-xia et al. (25)	N	Y	Y
Fang-yuan et al. (12)	Y	Y	Y
Caihong et al. (27)	Y	Y	Y
	3	4	4

Discarded article	Post-acupuncture > Pre-acupuncture								
	Posterior lobe of the cerebellum_L	PUT_R	MFG_R	IFG_R	PCUN_L	MTG_R	CLA_L	Cerebellar Tonsil_L	SMG_R
Xie et al. (20)	Y	Y	N	N	Y	N	Y	Y	Y
Xiaoqing (19)	Y	Y	Y	Y	N	N	Y	Y	Y
Yumei (22)	Y	Y	Y	Y	Y	Y	Y	Y	Y
Hinjing (21)	Y	Y	N	N	N	N	Y	Y	Y
Hinjing (21)	Y	Y	Y	Y	Y	Y	Y	N	N
Zhihong (23)	Y	Y	Y	Y	Y	Y	N	Y	N
Zhihong (23)	Y	Y	Y	Y	Y	Y	Y	Y	Y
Ping et al. (11)	Y	Y	Y	Y	Y	Y	Y	Y	N
Nana (24)	Y	Y	Y	Y	Y	Y	Y	Y	Y
Nana (24)	Y	Y	Y	Y	Y	Y	Y	Y	Y
Zhengfei et al. (26)	Y	N	Y	Y	Y	Y	N	Y	Y
Caihong et al. (27)	Y	Y	Y	Y	Y	Y	Y	Y	Y
Ximei et al. (28)	Y	Y	Y	Y	Y	Y	Y	N	N
Ximei et al. (28)	Y	Y	Y	Y	N	N	Y	Y	Y
Chen et al. (29)	Y	Y	Y	Y	Y	Y	Y	Y	Y
Jianzhong (32)	Y	Y	Y	Y	Y	Y	Y	Y	Y
	16	15	14	14	13	12	14	14	12

Discarded article	Post-acupuncture < Pre-acupuncture		
	MFG_R	PreCG_L	PCUN_R
Ximei et al. (28)	Y	Y	Y
Xiaoqing (19)	Y	Y	N
Yumei (22)	N	Y	Y
Zhihong (23)	Y	N	Y
Zhihong (23)	Y	Y	Y
Nana (24)	Y	Y	Y
Nana (24)	Y	Y	Y
Zhengfei and Rongai (26)	N	N	Y
Chen et al. (29)	Y	Y	Y
Jianzhong (30)	Y	Y	Y
	8	8	9

IFG, inferior frontal gyrus; PCUN, precuneus; PUT, lentiform nucleus putamen; MFG, medial frontal gyrus; MTG, middle temporal gyrus; CLA, claustrum; SMG, superior marginal gyrus; PreCG, precentral gyrus; R, right; L, left; Y, Yes; N, No.

## 4 Discussion

In this study, ALE analysis was used to integrate 14 original rs-fMRI studies to investigate the spontaneous brain activity in patients with motor dysfunction after stroke and the neurological effects of acupuncture on motor dysfunction after stroke. According to ALE analysis, spontaneous brain activity changes are likely to occur in the posterior lobes of the cerebellum, IFG, and PCUN in patients with motor dysfunction after stroke. After acupuncture treatment, active areas were the posterior lobe of the cerebellum, PUT, MFG, IFG, PCUN, MTG, CLA, SMG, and inactivated areas were MFG, PreCG, and PCUN.

The motor cortex is mainly located in the anterior central and anterior paracentral lobules (Brodmann [BA]4, BA6). The anatomy of the cerebral cortex the primary motor cortex (M1) is located at BA4 and is the higher center that controls motor activity (31). The premotor area (PMA) is located in the cortex before the central sulcus, in the anterior central gyrus and its anterior part, and is the nerve center that regulates the voluntary movement of the body (32). The auxiliary motor area (SMA) is located in front of the medial lateral para-central lobule of the cerebral hemisphere, and is a secondary motor area with the posteromedial cortex (PMC), which is located in BA6 (33). The fibers from SMA join the pyramidal tract and connect with M1 and PMC through the fibers to control and inhibit movement (34). The different motor functional areas of the cerebral cortex form complex interactive network connections and coordinate with each other to complete various movements. Multiple studies have shown that the above regions are involved in exercise preparation and execution, and that changes in their activity contribute to reorganization after stroke (35, 36). Our results showed that after acupuncture treatment, both BA4 and BA6 showed decreased neuronal activity. This is highly consistent with the results of a meta-analysis (14), and we hypothesize that this phenomenon is related to competitive compensatory activity between hemispheres (37, 38). The decrease of BA4 and BA6 activity may indicate the decrease of self-compensatory dependence, indicating the restoration of self-motor network, and thus contributing to the recovery of the affected limb. Since the focal areas included in the literature in this study are inconsistent, further experiments are needed to explore the compensatory mechanism of the motor cortex. In addition, feedback loops between M1, PMC and basal ganglia nuclei affect the activity of the pyramidal system. It is commonly believed that the basal ganglia is associated with dystonia and motor control in humans, and their damage can cause muscle contraction and relaxation disorders, resulting in certain technical movements that cannot be completed (39–41). The results of this study showed that the neuronal activity of the lentiform nucleus and the plateform nucleus in the basal ganglia of the brain increased significantly after acupuncture treatment. It is inferred that the limb motor dysfunction of ischemic stroke patients recovered from acupuncture is mainly related to the activation of basal ganglia. This is consistent with the results of another meta-analysis (14).

Our experimental results showed that in addition to the key components of the sensorimotor network M1, PMA and SMA mentioned above, the activity of PCUN, MFG, MTG, SMG, IFG and other brain regions also changed after acupuncture treatment. MTG is a key node in the default mode network (DMN), while MFG participates in the executive control network (ECN) (42). This suggests that acupuncture restoration of movement involves other brain networks besides SMN. A study has shown that the synchronization of DMN and sensorimotor network can promote the recovery of movement in stroke patients, acupuncture can regulate SMN, relieve hemiplegia, numbness and other symptoms (43–46). Meanwhile, the enhancement of MTG activity was positively correlated with the increase of Modified Barthel Index (MBI) score (44). Of course, acupuncture also has the therapeutic effect of improving cognitive ability and sensorimotor function by enhancing the connection between the cerebellum and the ECN. The precuneus is a deactivated brain region with the highest metabolic rate in the default network of the brain. It is not only indirectly connected with the cerebellum, but also has fiber connections with the M1 region. The results of this study showed that compared with healthy patients, the activity of the anterior cuneus was enhanced, which may be related to the decreased activity of the affected side due to ischemia and hypoxia, while the healthy side compensated, which was consistent with the results of Liu Dinghua et al. (47). In addition, we also found that the activity of the contralateral precuneus decreased after acupuncture, which may be due to the gradual recovery of motor function and the gradual weakening of compensatory range. Previous studies have confirmed that acupuncture is more likely to enhance spontaneous neural activity in DMN (48). The inconsistent results in this study may be due to the fact that the location of infarcts in stroke patients is not controlled, and the results obtained are highly biased, which is also the direction of our follow-up efforts.

The cerebellum is located at the back of the brain, covering the pons and medulla oblongata, and straddling between the midbrain and medulla oblongata. It has rich afferent and efferent connections with the cerebrum, brainstem and spinal cord, and is an important node of the motor network, mainly maintaining body balance, regulating muscle tension, coordinating muscle movement and maintaining posture (49, 50). In addition, control of movement is also a recognized role of the cerebellum, including learning motor coordination and posture and proficiency in movement. Studies have reported deficits in learning motor skills in patients with cerebellar lesions (51–53). Interestingly, we found that compared with healthy people, the posterior cerebellar lobe of the patients with motor dysfunction after stroke showed activation, and this brain area remained activated after acupuncture. We hypothesized that patients need to learn simple movement posture after stroke, and this learning process is dominated by cerebellar participation, so it is manifested as enhanced cerebellar nerve activity. After acupuncture, cerebellar activity was further enhanced, improving patients’ learning ability and promoting the reconstruction of motor function. In addition, relevant studies have shown that with the gradual recovery of motor function, motor relearning is gradually reduced, and cerebellar activity is also decreased (54).

## 5 Strengths and limitations

In this study, the results of acupuncture in motor dysfunction after stroke are controversial, and the coordinate based ALE method is innovatively adopted to conduct a secondary analysis of their findings. This analysis method prevents the atypical results in most studies from showing up in the results of this meta-analysis, greatly reducing the risk of false positive results, providing an important basis for the promotion of these findings, and revealing the possible neural mechanism of acupuncture in the treatment of post-stroke motor dysfunction. However, this study also has some limitations. Although strict inclusion and exclusion criteria were adopted in this study, there was still considerable heterogeneity among the studies included in the meta-analysis. Although the use of ALE methods significantly reduces the risk of false positive results, we still need to consider the impact of these heterogeneity on the results of the meta-analysis. This meta-analysis included studies with different statistical thresholds, different acupuncture parameters (frequency, referring to different acupuncture points, manipulation methods and timing), and uncertainties in the inclusion of patient encephalopathy, although this study also considered controlling for these possible biases. But this paper argues that it is more appropriate to include these studies with a certain risk of false-positive results than to ignore them entirely. Therefore, in order to better understand the neurological effects of acupuncture on post-stroke functional dysfunction, it is time to organize large-scale and high-quality clinical studies for further exploration.

## 6 Conclusion

Acupuncture can affect the activity of motor related areas, basal ganglia area, cerebellum and regions associated with DMN, SMA and ECN, which may be a potential mechanism for acupuncture therapy to restore motor function in stroke patients.

## Data Availability

The original contributions presented in the study are included in the article/Supplementary material, further inquiries can be directed to the corresponding author.

## Footnotes

^1^ https://training.cochrane.org/handbook
