# Supplementary material for: Neural effects of acupuncture on stroke patients with motor dysfunction: an activation likelihood estimation meta-analysis
Source: Front Neurol. 2024 Sep 25;15:1453935. doi: 10.3389/fneur.2024.1453935 (PMC11461336; doi:10.3389/fneur.2024.1453935)
Supplement: Supplementary file 1 [file Data_Sheet_1.docx]

**Neural effects of Acupuncture on Stroke Patients with Motor Dysfunction: An activation likelihood estimation meta-analysis**

**Supplementary Materials The full search strategies of all databases**

| **Database** | **Coverage** | **Searches** | **Hits** |
| --- | --- | --- | --- |
| PubMed | Date of inception -March 21, 2024 | (((("Stroke"[Mesh]) OR (((((((((((((Cerebrovascular) OR (Poststroke)) OR (Cerebrovascular Accident)) OR (CVA)) OR (Cerebrovascular Apoplexy)) OR (Brain Vascular Accident)) OR (Cerebrovascular Stroke)) OR (Apoplexy)) OR (Cerebral Stroke)) OR (Acute Stroke)) OR (Acute Cerebrovascular Accident)) OR (hemipleg)) OR (Cerebral Infarction))) AND (((((acupuncture) OR (electroacupuncture)) OR (needle)) OR (acupoint)) OR (pharmacopuncture))) AND ((((((fMRI) OR (functional MRI)) OR (functional magnetic resonance imaging)) OR (neuroimaging)) OR (Resting-state functional magnetic resonance)) OR (rs-fmri))) | 366 |
| EMBASE | Date of inception -March 21, 2024 | #1:'stroke/exp OR stroke OR cerebrovascular OR poststroke OR 'cerebrovascular accident/exp OR 'cerebrovascular accident OR (cerebrovascular AND ('accident'/exp OR accident)) OR 'cva'/exp OR cva OR 'cerebrovascular apoplexy' OR (cerebrovascular AND ('apoplexy'/exp OR apoplexy)) OR 'brain vascular accident/exp OR 'brain vascular accident' OR (('brain'/exp OR brain) AND vascular AND ('accident'/exp OR accident) OR 'cerebrovascular stroke OR (cerebrovascular AND (stroke'/exp OR stroke)) OR 'apoplexy'/exp OR apoplexy OR 'cerebral stroke'/exp OR 'cerebral stroke' OR (cerebral AND ('stroke'/exp OR stroke)) OR 'acute stroke'/exp OR 'acute stroke' OR (acute AND (stroke'/exp OR stroke)) OR 'acute cerebrovascular accident OR (acute AND cerebrovascular AND ('accident'/exp OR accident)) OR hemiple OR 'cerebral infarction'/exp OR 'cerebral infarction' OR (cerebral AND ('infarction'/exp OR infarction))  #2: acupuncture OR electroacupuncture OR needle OR acupoint OR pharmacopuncture  #3:((fmri OR functional) AND mri OR functional) AND magnetic AND resonance AND imaging OR neuroimaging OR 'resting state') AND functional AND magnetic AND resonance OR 'rs fmri*  #4: #1AND #2 AND #3 | 250 |
| Web of Science | Date of inception -March 21, 2024 | #1: TS=( stroke OR Cerebrovascular OR Poststroke OR Cerebrovascular Accident OR CVA OR Cerebrovascular Apoplexy OR Brain Vascular Accident OR Cerebrovascular Stroke OR Apoplexy OR Cerebral Stroke OR Acute Stroke OR Acute Cerebrovascular Accident OR hemipleg OR Cerebral Infarction)  #2: TS=( acupuncture OR electroacupuncture OR needle OR acupoint OR pharmacopuncture)  #3: TS=(fMRI OR functional MRI OR functional magnetic resonance imaging OR neuroimaging OR Resting-state functional magnetic resonance OR rs-fmri)  #4: #3 AND #2 AND #1 | 233 |
| Cochrane Library | Date of inception -March 21, 2024 | #1: MeSH descriptor: [Stroke] explode all trees  #2: Cerebrovascular OR Poststroke OR Cerebrovascular Accident OR CVA OR Cerebrovascular Apoplexy OR Brain Vascular Accident OR Cerebrovascular Stroke OR Apoplexy OR Cerebral Stroke OR Acute Stroke OR Acute Cerebrovascular Accident OR hemipleg OR Cerebral Infarction  #3: MeSH descriptor: [Acupuncture] explode all trees  #4: #1 OR #2  #5: electroacupuncture OR needle OR acupoint OR pharmacopuncture  #6: #3 or #5  #7: MeSH descriptor: [Magnetic Resonance Imaging] explode all trees  #8: fMRI OR functional MRI OR functional magnetic resonance imaging OR neuroimaging OR Resting-state functional magnetic resonance OR rs-fmri  #9: #7 or #8  #10: #4 and #6 and #9 | 74 |
| China National Knowledge Infrastructure (CNKI, Chinese Database) | Date of inception -March 21, 2024 | TKA= ('卒中' + '中风' + '偏瘫' + '卒中后' + '脑梗塞' + '脑梗死' + '脑血管疾病' + '急性中风' ) * ( '针刺' + '针灸' + '电针' + '头针' + '针' ) * ('功能磁共振' + '磁共振成像' + '静息态功能磁共振' + 'fMRI' )) | 1154 |
| Chinese Biomedical Literature Database  (CBM, Chinese Database) | Date of inception -March 21, 2024 | ( "卒中"[常用字段:智能] OR "中风"[常用字段:智能] OR "偏瘫"[常用字段:智能] OR "卒中后"[常用字段:智能] OR "脑梗塞"[常用字段:智能] OR "脑梗死"[常用字段:智能] OR "脑血管疾病"[常用字段:智能] OR "急性中风"[常用字段:智能]) AND( "针刺"[常用字段:智能] OR "针灸"[常用字段:智能] OR "电针"[常用字段:智能] OR "头针"[常用字段:智能] OR " 针"[常用字段:智能]) AND( "功能磁共振"[常用字段:智能] OR "磁共振成像"[常用字段:智能] OR "静息态功能磁共振"[常用字段:智能] OR " fMRI"[常用字段:智能]) | 934 |
| China Science and Technology Journal Database (VIP, Chinese Database) | Date of inception -March 21, 2024 | 题名或关键词:(卒中 or 中风 or 偏瘫 or 卒中后 or 脑梗塞 or 脑梗死 or 脑血管疾病 or 急性中风) and 题名或关键词:(针刺 or 针灸 or 电针 or 头针 or 针) and 题名或关键词:(功能磁共振 or 磁共振成像 or 静息态功能磁共振 or fMRI) | 122 |
| Wanfang Database  (WF, Chinese Database) | Date of inception -March 21, 2024 | 题名或关键词:(卒中 or 中风 or 偏瘫 or 卒中后 or 脑梗塞 or 脑梗死 or 脑血管疾病 or 急性中风) and 题名或关键词:(针刺 or 针灸 or 电针 or 头针 or 针) and 题名或关键词:(功能磁共振 or 磁共振成像 or 静息态功能磁共振 or fMRI) | 159 |
